# Allied Health Professionals’ Perceptions of Artificial Intelligence in the Clinical Setting: Cross-Sectional Survey

**DOI:** 10.2196/57204

**Published:** 2024-12-30

**Authors:** Jane Hoffman, Laetitia Hattingh, Lucy Shinners, Rebecca L Angus, Brent Richards, Ian Hughes, Rachel Wenke

**Affiliations:** 1 Pharmacy Department Gold Coast Hospital and Health Service Southport Australia; 2 School of Pharmacy and Medical Sciences Griffith University Southport Australia; 3 School of Pharmacy University of Queensland Brisbane Australia; 4 Faculty of Health Southern Cross University Bilinga Australia; 5 School of Medicine and Dentistry Griffith University Southport Australia; 6 School of Medicine University of Queensland Brisbane Australia

**Keywords:** allied health, artificial intelligence, hospital, digital health, impact, AI, mHealth, cross sectional, survey, health professional, medical professional, perception, clinical setting, opportunity, challenge, healthcare, delivery, Australia, clinician, confirmatory factor analysis, linear regression

## Abstract

**Background:**

Artificial intelligence (AI) has the potential to address growing logistical and economic pressures on the health care system by reducing risk, increasing productivity, and improving patient safety; however, implementing digital health technologies can be disruptive. Workforce perception is a powerful indicator of technology use and acceptance, however, there is little research available on the perceptions of allied health professionals (AHPs) toward AI in health care.

**Objective:**

This study aimed to explore AHP perceptions of AI and the opportunities and challenges for its use in health care delivery.

**Methods:**

A cross-sectional survey was conducted at a health service in, Queensland, Australia, using the Shinners Artificial Intelligence Perception tool.

**Results:**

A total of 231 (22.1%) participants from 11 AHPs responded to the survey. Participants were mostly younger than 40 years (157/231, 67.9%), female (189/231, 81.8%), working in a clinical role (196/231, 84.8%) with a median of 10 years’ experience in their profession. Most participants had not used AI (185/231, 80.1%), had little to no knowledge about AI (201/231, 87%), and reported workforce knowledge and skill as the greatest challenges to incorporating AI in health care (178/231, 77.1%). Age (*P*=.01), profession (*P*=.009), and AI knowledge (*P*=.02) were strong predictors of the perceived professional impact of AI. AHPs generally felt unprepared for the implementation of AI in health care, with concerns about a lack of workforce knowledge on AI and losing valued tasks to AI. Prior use of AI (*P*=.02) and years of experience as a health care professional (*P*=.02) were significant predictors of perceived preparedness for AI. Most participants had not received education on AI (190/231, 82.3%) and desired training (170/231, 73.6%) and believed AI would improve health care. Ideas and opportunities suggested for the use of AI within the allied health setting were predominantly nonclinical, administrative, and to support patient assessment tasks, with a view to improving efficiencies and increasing clinical time for direct patient care.

**Conclusions:**

Education and experience with AI are needed in health care to support its implementation across allied health, the second largest workforce in health. Industry and academic partnerships with clinicians should not be limited to AHPs with high AI literacy as clinicians across all knowledge levels can identify many opportunities for AI in health care.

## Introduction

Artificial intelligence (AI) has been hailed as a solution to address the growing logistical and economic pressures on the health care system due to an aging population, rising chronic disease burden, and workforce shortages [1,2]. AI is a term used to describe a large and growing range of computer functions that can “learn” from data to make better decisions over time, such as machine learning, natural language processing, and computer vision [3]. The potential of AI in health care lies in its ability to analyze unstructured data, detect abnormalities, provide correlations, and automate or assist with some human tasks [4]. Although the implementation of digital health including AI technologies can initially be disruptive [5,6] it may be useful to improve work productivity and clinical workflow, reduce risk and error, and augment clinical decision-making, which would ultimately improve patient safety and outcomes such as document summarization [7-9].

Until recently, research about AI has been disproportionally focused on the merits of the technology, while investigation into workforce readiness and preparation for this new generation of technology is limited [10,11]. Lessons from previous industrial revolutions show that successful technology implementation is directly dependent on understanding and acknowledging the social dimensions of the human-technology relationship [12]. The more complex the technology and the setting such as AI in health care, the less likely it is to be successfully adopted by the people intended to use it [13,14]. It is anticipated that in the future, digital health will be practiced by the same health care professionals who currently deliver traditional care. It is not yet known what the full impact of digital technology will be on the health care industry [15]. It is essential to understand and address the perceptions of health care professionals toward AI, as negative attitudes can lead to health technology abandonment, nonadoption, and misuse, which ultimately negatively impacts efforts to improve patient safety and quality of care [14,16,17].

Advancing digital capability in allied health (AH) is one strategy used to address the increasing demands on a public health service in Queensland, Australia [18,19]. Technology implementation improves when health care professionals understand the purpose of the technology, how it is used, and how relevant it is to their role [2]. In addition, the ease of implementation and ultimately the acceptance of technology relies on early and sustained user engagement [20,21]. Committed to maintaining and advancing further digital capability [18], our digital health service identified a need to understand allied health professionals’ (AHPs) perceptions of AI to help prepare staff for future AI implementations. Workforce perception is a powerful indicator of organizational readiness and is a predictor of technology use and acceptance [10,20]. Research on health care professionals’ perceptions of AI is emerging as organizations seek to understand workforce readiness [2,22-24]. Currently, most of this research has explored the perceptions of medical professionals and nurses [16,23,25,26]. AH literature has focused on professions such as radiology [27,28], medical imaging [29,30], and pharmacy [31-34] with some research on physiotherapy [35,36] and audiology [37] that may reflect more advanced stages of AI adoption in those professions. While these studies provide valuable insights into how these professions can be supported in using and implementing AI, few studies capture the perceptions of a variety of AHPs to obtain insights into their perceptions and readiness and how they compare with each other [16,38]. No internationally agreed definition of AH exists, yet the core functions and types of AH roles are similar between countries, although there may be some differences in education and the scope of practice [38]. Australian AHPs are university-qualified practitioners with accredited, specialized expertise working within a set scope of practice to prevent, diagnose, and treat a range of conditions and illnesses [39-41]. AH is the second largest health workforce in Australia consisting of at least sixteen diverse professions such as physiotherapy, pharmacy, occupational therapy, speech pathology, and dietetics among others [39-46].

The professional skills, knowledge, work practices, and patient contact are heterogenous for AHPs [45], which differ from the homogenous attributes described for nursing [46] and medical professionals [44,45]. This highlights the need to better understand the perception of AHPs as a group while also exploring any differences between AH professions. There is great potential for AI technology in many AH professions, with emerging technologies such as clinical decision support, wearable technologies, adverse drug reaction, drug interaction detection, and providing health information and advice [34,47,48]. However, a human-centered understanding of the AHP workforce characteristics and perceptions is needed to ensure successful implementation and adoption of AI technology [49-51].

To investigate the perceptions of AHP on AI per its increased role in the workplace we used the Shinners Artificial Intelligence Perception (SHAIP) [52] tool. SHAIP was designed in 2019 due to an Australian e-Delphi study [53], which gathered the opinions of an interdisciplinary panel of experts in health and technology. It is underpinned by the sociotechnical systems theory, which acknowledges the complex relationships between the individual, the technology, and the workplace and supports the belief that organizations need a human-centered understanding of workforce characteristics and perceptions when implementing new technology [54]. The tool was tested and validated in 2021 [53]. The SHAIP tool is a 10-item, 2-factor tool that measures health care professionals’ perceptions of the professional impact of AI and preparedness for AI [52]. Literature using the SHAIP tool [52] is emerging worldwide, however only a small number of AHPs have been represented in the results [23,24]. Knowledge of AHPs’ perceptions of AI and the opportunities they envision for their disciplines will be valuable to both organizations and industry, facilitating research targeted toward AI interventions that create clinical efficiencies in the AH setting.

To our knowledge, this project is the first of its kind to exclusively investigate AHP perceptions of AI and the opportunities and challenges for its use in health care delivery.

## Methods

### Study Design

A cross-sectional survey that included SHAIP was conducted in mid-2023 at Gold Coast, Queensland, Australia, with AHPs employed across one large tertiary hospital and health service. The STROBE (Strengthening the Reporting of Observational Studies in Epidemiology) guidelines were followed to report our study’s findings [55]. The survey study was part of a larger project that also incorporated qualitative data collection through focus groups with AH clinicians and managers, a paper entitled “Overcoming barriers and enabling artificial intelligence adoption in allied health clinical practice: a qualitative study” is under preparation to be published elsewhere.

### Ethical Considerations

Ethics approval was granted by the Gold Coast Health Human Research Ethics Committee (HREC/2023/QGC/96821). Participant consent was provided via acceptance of a consent statement on the opening page of the survey. The survey was anonymous, with an option for participants to provide contact details to participate in further parts of the project, which was kept separate from the collected data. Any identifiable data was removed from collected data and stored on a limited access drive, separate from this study’s data that is only accessible by research team members. No compensation or reimbursement was offered to participate in this study.

### Study Setting and Participants

The Gold Coast Hospital and Health Service (GCHHS) employs approximately 10,000 staff to deliver public health care services to a general population of over 630,000 [56-58]. Approximately 1200 AHPs are employed at GCHHS across two tertiary hospitals, one day surgery hospital, health precincts, and community health services [58]. Eleven AH departments support 16 AH professions [59] and were listed in the data collection tool, with an “other” option available to AHP not represented by these areas. All GCHHS AHPs were eligible to participate in this study [58].

### Study Recruitment

An online survey using Microsoft Forms that included the SHAIP tool was disseminated to AHPs at GCHHS via email. A link to this study was also advertised via online broadcasts, posters, and staff meetings. The survey was open for a period of seven weeks from May 17 to July 6, 2023.

### Study Measures

This study design was adapted from the study conducted by Shinners et al [24] with the addition of locally developed questions. The survey was piloted on 5 AHPs to test face and content validity, which resulted in minor changes to wording [60,61]. Participant demographics were collected, including age group, sex, facility (Gold Coast University Hospital, Robina Hospital, Varsity Lakes Day Hospital, health precinct, or community health), AH profession (audiology, clinical measurements, dietetics, medical imaging, occupational therapy, pharmacy, physiotherapy, podiatry, psychology, speech pathology, or social work), role (clinical informatics or technology, clinician, educator or clinical facilitator, governance, manager, researcher, or academic), years of experience in profession, years of experience using the “integrated electronic medical record,” AI knowledge (no knowledge, beginner understanding, intermediate understanding, or advanced understanding), and previous use of AI (yes, no, or unsure). Participants were asked to complete the 10-item, 2-factor SHAIP tool [19] that measures health care professionals’ perceptions of factor one: professional impact of AI, and factor two: preparedness for AI. These questions sought participant agreement along a five-point Likert scale rating (1 totally agree to 5 totally disagree) with a neutral point of 3 “unsure.”

Finally, participants indicated any prior AI education they had received (none, self-initiated online course, webinar, conference, workplace training, or formal university qualification), if they would like to receive AI education (yes, no, or unsure), and what type of AI education they would like to receive (general teaching about AI capabilities, the application of AI in health care, or the ethics of AI in health care) from a list adapted from Shinners et al [24]. Participants were also asked to identify challenges that exist for AI implementation (infrastructure, interoperability with current systems, cost to implement, workforce knowledge and skills, organizational support, interdisciplinary collaboration, clinical governance, research funding, change fatigue, workforce resilience, or “I don’t know”). Using open-ended questions, participants were asked to describe their understanding of AI, and ideas or opportunities for AI that could be developed or implemented in their current practice.

### Data Analysis

Completed responses were imported into Stata (version 17; StataCorp LLC). Demographic data, including age group, sex, and years of experience, were collected. Descriptively, the responses to each Likert scale question were presented as a median and IQR. Confirmatory factor analysis (CFA) was conducted to verify the 2-factor model reported by Shinners et al [52] that identified factor one: professional impact of AI, and factor two: preparedness for AI as factors. A structural equation modelling approach was used to conduct the CFA and standardized coefficients (loading factors) were produced. We initially used all 10 questions in the SHAIP tool with questions 1 to 5 and 7 expected to load onto factor one and questions 6, 8, 9, and 10 to load onto factor two as suggested by Shinners et al [52]. Model goodness-of-fit was initially assessed by observation of individual question coefficients, their *P* values and *R*^2^ values. Global model goodness-of-fit measures considered were the chi-square test (model vs saturated; though considered over sensitive in large sample sizes), the comparative fit index (CFI) and Tucker-Lewis index (TLI), the root-mean-square error of approximation (RMSEA), and the standardized root-mean-squared residual (SRMR). Values >0.90 for the CFI and TLI were considered indicators of a good fit. Values of 0.05 to 0.08 for RMSEA and 0.05 to 0.10 for SRMR were considered indications of acceptable fit. A test of acceptable fit based on the RMSEA, the PCLOSE test, was also performed. We also used modification indices (*estat minidices* command of Stata) to identify possible model respecifications to improve fit.

Factor scores for the professional impact of AI and preparedness for AI factors were calculated for each individual based on the final structural equation modeling model. These factor scores are similar to *z* scores with 0 as the mean and values being the number of SDs from the mean with more negative values indicating more improvement or agreement. Simple factor scores equal to the mean Likert scale value of the questions contributing to the factor were also calculated. Cronbach α was calculated to estimate the internal consistency of each factor given the loading questions and structural equation modelling model.

Linear regression was used to identify predictors of each of the 2 factors (as factor scores) identified via CFA: professional impact of AI and preparedness for AI. Potential predictor variables considered were AH profession, age group, sex, AI knowledge, current use of AI, and years of experience in their profession. Initially, variables were considered in isolation and included in a multivariable model if there was evidence of an effect on professional impact of AI or preparedness for AI (*P*<.10). Variables were retained in the model if *P*<.05. Different models were constructed using factor scores or simple Likert scale values. The effects of predictor variables are presented as adjusted mean factor scores (AMFS) or approximate adjusted mean Likert scale (AAMLS) values. Differences between AH professions concerning professional impact of AI and preparedness for AI were further investigated by identifying professions that differed from the grand mean after adjustment for multiple comparisons (*P*<.05) by the Sidak method. Pairwise comparisons between each profession were also performed by 1-way ANOVA and the post hoc Fisher–Hayter pairwise comparisons procedure, which adjusts for multiple comparisons by calculating the critical value of the studentized range (CVSR) above which differences have a *P*<.05.

Open-ended questions defining AI were deductively grouped into four categories using NVivo (QSR International) according to the framework described in Shinners et al [24]. Categories were then iteratively revised by the research team and category definitions refined.

Inductive content analysis was used to group responses to open-ended questions on ideas or opportunities for AI that could be developed or implemented in current practice. The major categories and subcategories identified were refined by the research team to reach a consensus.

## Results

### Participant Demographics

GCHHS human resources management confirmed that 1045 AHPs were employed at GCHHS at the time of this study. A total of 245 participants completed the survey. Fourteen responses were removed: 8 due to incomplete data (participants had not answered any questions) and 6 as participants were not AHPs. A final 231 responses remained representing 22.1% (231/1045) of the total population. Respondents were predominantly younger than 40 years (157/231, 67.9%), female (189/231, 81.8%), primarily working in a clinical role (196/231, 84.8%), with a median of 10 years (IQR 6 to 17) experience in their discipline, and working mostly at one hospital (186/231, 80.5%; Table 1). Eleven AH professions were represented in the data, with the majority being pharmacists (46/231, 19.9%), physiotherapists (39/231, 16.9%), and occupational therapists (38/231, 16.5%). The AH departments with the highest response rate were audiology (5/8, 62.5% of all audiologists) and dietetics (29/64, 45.3% of all dietitians). Conversely, only 8.5% (11/128) of medical imaging staff responded and no clinical measurement staff completed the survey. Most respondents reported they were not using AI in their current role (185/231, 80.1%) and most (201/231, 87.0%) rated their knowledge of AI as either beginner or having no knowledge. More than three-quarters of respondents believed workforce knowledge and skill were the greatest challenge to incorporating AI in health care (178/231, 77.1%), followed by infrastructure (141/231, 61.0%) and workforce resistance (119/231, 51.5%).

**Table 1:** Participant demographics (N=231).

Question and category			Participants, n (%)
Age (years)			
	18-30	58 (25.1)	
	31-40	99 (42.9)	
	41-50	43 (18.6)	
	51-60	27 (11.7)	
	61-70	4 (1.7)	
Sex			
	Man	40 (17.3)	
	Woman	189 (81.8)	
	Prefer not to say	2 (0.9)	
	Another term	0 (0)	
Profession			
	Audiology	5 (2.2)	
	Clinical measurements	0 (0)	
	Dietetics	29 (12.6)	
	Medical imaging	11 (4.8)	
	Occupational therapy	38 (16.5)	
	Other or unknown	5 (2.1)	
	Pharmacy	46 (19.9)	
	Physiotherapy	39 (16.9)	
	Podiatry	3 (1.3)	
	Psychology	11 (4.8)	
	Social work	27 (11.7)	
	Speech therapy	17 (7.4)	
Role			
	Clinical informatics or technology	5 (2.2)	
	Clinician	196 (84.8)	
	Educator or clinical facilitator	5 (2.2)	
	Governance	1 (0.4)	
	Manager	18 (7.8)	
	Researcher academic	4 (1.7)	
	Unknown	2 (0.9)	
Site (multiselect option)			
	Gold Coast University Hospital	186 (80.5)	
	Robina	93 (40.3)	
	Varsity Lakes	5 (2.2)	
	Health precinct or community health	37 (16)	
	Other or unknown	3 (1.3)	

### About CFA

Initial CFA using the original SHAIP tool showed that question 10 “I believe that should AI technology make an error, full responsibility lies with the healthcare professional” had a low correlation (0.097, *P*=.26) with the preparedness for AI factor. The CFI was marginally above the acceptable criteria (0.905>0.90), the TLI was below the acceptable cutoff (0.87<0.95) and the RMSEA was 0.8, being at the margin of acceptable (PCLOSE test of RMSEA <0.05, *P*=.01), and chi-square *P*=3.5×10^–6^. Taken together, this suggested the model was not a good fit for the data sample.

A revised model was created in which question 10 was removed and a correlation between question 4 and question 7 was included in the model following suggestions from assessment of modification indices.

The revised, 9-item model was reanalyzed using CFA. In this model, CFI=0.966, TLI=0.955, RMSEA=0.048 (PCLOSE *P*=.52), SRMR=0.067, and chi-square *P*=.02. Although the chi-square remained significant, all other measures indicated a good fit. Cronbach α was used to determine the reliability of the two factors. A Cronbach α score >0.7 indicates good internal consistency however a Cronbach α of 0.5 or 0.6 can be used in some cases [62-64]. The professional impact of AI factor had a Cronbach α of 0.82 while the preparedness for AI factor had a lower reliability with Cronbach α=0.54, which is likely due to the small number of items contributing to the factor [62].

### Factors Influencing Perceptions of AI Using the SHAIP Tool

#### Overview

**Figure 1:**
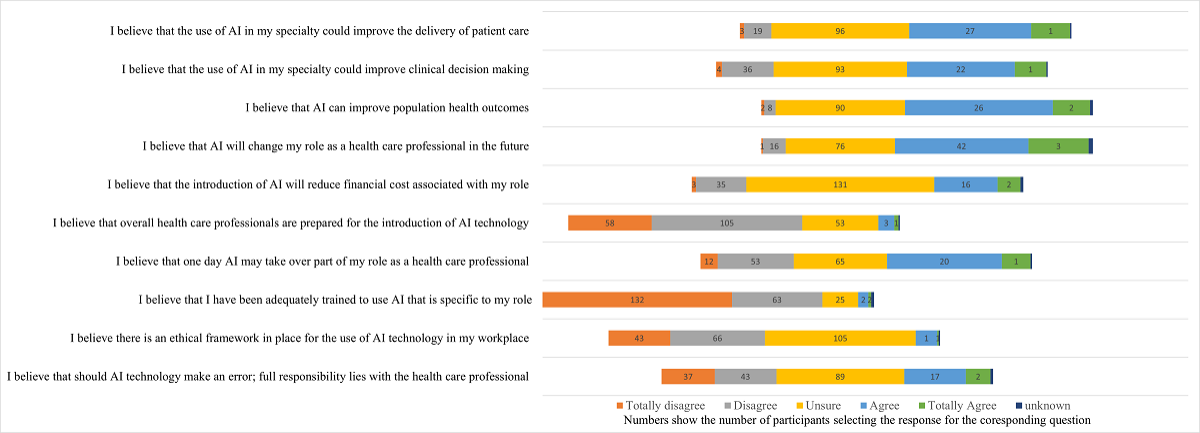
AHP responses to SHAIP questions about perceptions of AI in health care (N=231). AHP: allied health professional; AI: artificial intelligence; SHAIP: Shinners Artificial Intelligence Perception.

Likert scale responses were analyzed for overall perceptions of AI for each question (Figure 1). This analysis showed that AHPs generally agreed with the statements relating to the use of AI in their specialty “could improve the delivery of patient care,” “improve clinical decision making,” “improve population health outcomes,” and that AI will “change my role as a healthcare professional in the future.” Participants were less confident about the statements relating to AI reducing “financial costs” and AI taking over “part of my role as a healthcare professional.” In contrast, participants disagreed with the statements “healthcare professionals are prepared for the introduction of AI technology,” and that there is an “ethical framework in place” for AI, or that “should AI technology make an error; full responsibility lies with the healthcare professional.” AHPs totally disagree with the statement that they are “adequately trained to use AI.”

Participant responses were stronger when relating to the individual, and less sure if statements related to the overall profession. For example, 84.4% (195/231) of participants disagreed with the statement “I believe that I have been adequately trained to use AI that is specific to my role” (strongly disagree 132/231 or disagree 63/231) compared to 45.5% (105/231) being unsure about the statement “I believe overall healthcare professionals are prepared for the introduction of AI technology.”

#### Factor One: Professional Impact of AI

Multivariable linear regression identified that age group, profession, and AI knowledge were independent predictive factors for AHPs’ perception of the professional impact of AI. AHPs aged 51-60 years group were more likely to disagree that AI would affect their professional role (AMFS=0.24; AAMLS=2.92) than other age groups (AMFS=–0.032; AAMLS=2.56, *P*=.01). For profession, pharmacists were most likely to think AI would affect their professional role compared to other AH disciplines (AMFS=–0.314; AAMLS=2.22, *P*=.01), with physiotherapists (AMFS=0.215; AAMLS=2.90, *P*=.12) and social workers (AMFS=0.189; AAMLS=2.91, *P*=.42) being the least likely. ANOVA (unadjusted) using the Fisher-Hayter pairwise comparison post hoc analysis adjustment for multiple comparisons at the *P*<.05 significance level calculated the CVSR to be 4.60. Thus, occupational therapists (CVSR=5.56), physiotherapists (CVSR=7.97), and social workers (CVSR=7.40) were less likely than pharmacists (*P*<.05) to think AI would affect their professional role. No other differences with *P*<.05 were identified between professions.

Perceived AI knowledge was shown to influence a person’s agreement on the professional impact of AI (*P*=.02). People with advanced knowledge (AMFS=–0.488; AAMLS=1.94) were more likely to agree AI would have an impact compared to those with an intermediate understanding (AMFS=–0.086; AAMLS=2.58, *P*=.10), a beginner understanding (AMFS=–0.014; AAMLS=2.59, *P*=.04), or no knowledge (AMFS=0.166; AAMLS=2.76, *P*=.007).

#### Factor Two: Preparedness for AI

Overall, it was apparent from the mean Likert scale score (4.00) that very few AHPs felt prepared for AI (only 5 of 231, AAMLS≤3.00). Multivariable linear regression identified that current use of AI was an important independent predictive factor for preparedness for AI (*P*=.04). People who did not currently use AI were more likely to disagree that they were prepared for AI (AMFS=0.55; AAMLS=4.02) than people who were unsure that they were using AI (AMFS=–0.258; AAMLS=3.68, *P*=.02) or who confirmed they were currently using AI (AMFS=–0.140; AAMLS=3.68, *P*=.29). Years of experience in a profession was also a predictor for preparedness for AI (*P*=.02) such that the factor score increased by 0.012 (or approximately 0.009 of a Likert Scale) per year of experience. That is, more experienced AHP felt less prepared. No other predictive factors were identified for AI preparedness for AI as shown in Table S1 in Multimedia Appendix 1.

### Perceptions of Education

Participants indicated what type of AI education they had previously received and what type of education they required. Most participants indicated that they had not received any education on AI (190/231, 82.3%; Table 2). As shown in Table 2 most participants (194/231, 84%) perceived “application of AI” as the area of education most required. “Ethics of AI” (158/231, 68.4%) and “general teaching about AI” (156/231, 67.5%) demonstrated a similar rating, while “AI techniques” (131/231, 56.7%) was selected by more than half the participants.

**Table 2:** Descriptive analysis of AI^a^ education, experience, and barriers (N=231).

Question and category			Participants, n (%)
How do you rate your understanding of AI?			
	Advanced knowledge	5 (2.2)	
	Intermediate understanding	25 (10.8)	
	Beginner understanding	160 (69.3)	
	No knowledge	41 (17.7)	
What education or training have you had about AI?			
	None	190 (82.3)	
	Self-initiated online course, webinar, conference	32 (13.9)	
	Other	11 (4.8)	
	Workplace training	5 (2.2)	
	Formal university qualification	2 (0.9)	
Would you like to receive education about AI?			
	Yes	170 (73.6)	
	Unsure	41 (17.7)	
	No	19 (8.2)	
	Unknown	1 (0.4)	
Which AI topics would you like to know more about?			
	The application of artificial intelligence in healthcare	194 (84)	
	The ethics of artificial intelligence in healthcare	158 (68.4)	
	General teaching about artificial intelligence capabilities	156 (67.5)	
	Training on artificial intelligence techniques	131 (56.7)	
	Unknown	20 (8.7)	
	Other	5 (2.2)	
How many years’ experience with ieMR^b^?			
	0	3 (1.3)	
	0-1	11 (4.8)	
	1-2	26 (11.3)	
	2-3	21 (9.1)	
	3-4	42 (18.2)	
	4+	126 (54.5)	
	Unknown	2 (0.9)	
In your current role are you using AI?			
	Yes	14 (6.1)	
	Unsure	32 (13.9)	
	No	185 (80.1)	
	Unknown	0 (0)	
Challenges to incorporating AI in your workplace?			
	Infrastructure	141 (61)	
	Interoperability with current systems	117 (50.6)	
	Cost to implement	118 (51.1)	
	Workforce knowledge and skills	178 (77.1)	
	Organizational support	89 (38.5)	
	Interdisciplinary collaboration	62 (26.8)	
	Clinical governance	112 (48.5)	
	Research funding	61 (26.4)	
	Change fatigue	83 (35.9)	
	Workforce resistance	119 (51.5)	
	I don’t know	19 (8.2)	
	Other	38 (16.5)	

^a^AI: artificial intelligence.

^b^ieMR: integrated electronic medical records.

### Understanding of AI

For the question “in your own words, what do you understand artificial intelligence to mean,” 21 participants either did not respond or indicated they did not know. The remaining 210 participants provided a total of 220 responses, which were allocated into one of four categories.

Almost half of the participants (96/220, 44%) defined AI as computers with intelligence. For example: “computer-generated intelligence,” “intelligence created by a machine as opposed to a human,” and “use of intelligent/intuitive technology.”

Over a third (79/220, 36%) of participants defined AI as equipment with nonhuman qualities. Statements included*:* “computer programs,” “using technology,” and “computer generated outcomes based on algorithms.”

Eleven percent (25/220) of responses defined AI as having human-like qualities with statements such as “computers being able to think for themselves,” “computers and software with the ability to think and learn independently,” and “an online personality.”

Four percent (9/220) defined AI as a robot with statements such as “robots and other assistive tech devices,” “robotics,” and “robots.”

Eleven responses were unable to be categorized into the framework. Such statements included “ability to imitate” and “AI may enhance our work in the future, however, without regulation the risks are high for incorrect information.”

Further analysis revealed a theme expressing concern about the loss of tasks or occupations due to the introduction of AI. Fourteen percent (30/220) described it as “basically a way to take away jobs from humans,” “taking human interaction or judgement out of the equation,” and “preprogrammed intelligence that has capacity to attempt to interact as a human replacement.”

### Opportunities for AI in AH

Seventy participants responded to the open-ended question to describe ideas for AI opportunities, providing a total of 90 ideas. Responses were grouped into 6 categories. “Administrative” included opportunities for AI in staff rostering, referral management, correspondence, and documentation. “Clinical decision support” listed ideas in patient monitoring, screening, and assessment along with risk stratification and prioritization. “Medication management” identified medication reconciliation, adherence, and optimization tasks plus medication information solutions for patients. “Educational” opportunities included clinician simulation and training and novel patient education resources. “Treatment support” identified ideas for AI such as obtaining and synthesizing patient history collateral and wearable technologies improving access to health care. “Auditing and analytics” revealed opportunities in workplace auditing, improving workflow efficiencies, and analyzing population trends to predict demand.

## Discussion

### Principal Findings

This study explored AHP perceptions of AI in health care provision as well as the opportunities and challenges for its use in health care delivery in a large tertiary health service. The findings reveal that although the lack of AI knowledge and skills are the greatest barriers to AI implementation in health care, AHPs remain optimistic about the potential benefits of AI on health care and desire AI training and education. Key factors that influence AHP perceptions of AI were identified, such as the AH profession and the use of AI. Leveraging these factors could help inform future implementation strategies. Although most participants had limited AI knowledge, they were able to identify opportunities for AI in the delivery of health care. This study is the first to our knowledge that has exclusively explored AHP perceptions, providing unique insights that may inform future workforce readiness and education initiatives as well as guide further research and implementation strategies.

### Factors Influencing Perceptions of AI

This study showed that age, profession, and AI knowledge are key predictors of perceptions about the professional impact of AI, while the use of AI and years of experience in the profession were predictors of perceptions about preparedness for AI.

A prior study identified age as an important variable influencing digital transformation in health care, reporting that younger health professionals thought it was too slow when compared to older participants [65]. Interestingly, this study found that AHPs aged 51 to 60 years were less likely to perceive that AI would affect their professional role compared to younger age groups. Similarly, one Australian study [66] found that more experienced medical officers (>30 years of experience) were less likely to expect AI would impact their role in the coming decade [66]. However, evidence regarding the influence of age on perceptions of AI in health care has been varied and inconclusive [16,24,52]. Further, this study also found that more experienced AHPs perceived they were less prepared for AI, which has not been identified in prior studies [23,24]. The influence of age and experience on AHP perceptions may be explained by how imminent clinicians perceive AI in health care. The average age at retirement in Australia is 56.3 years, and people who are currently working intend to retire at 65.5 years of age [67]. If AHPs aged between 51 and 60 years do not believe AI will be introduced in the next 10 years or more, they may intend to retire before they expect the digital revolution will occur; therefore, they will not be affected by AI in health care as an AHP.

This is inconsistent with the global and domestic strategic planning to support the surge of AI and digital health technologies in health care currently rather than in 10 years [2,8,18,68]. Communicating expected timeframes of AI in health care may help AHPs of all ages and experience to be more aware of the likely immediacy of the change.

AHP perceptions on the professional impact of AI varied based on individual AH professions This may be due to the extent that each AH profession uses technology to deliver health care. Radiology [27,28] and medical imaging [29,30] are data-rich professions, leading AI adoption in AH care and more likely to use data-driven innovation than other AHPs such as social work. Pharmacists in this study were more likely to perceive an impact on their professional role compared to occupational therapists, physiotherapists, and social workers. The emerging evidence relating to the application of AI in pharmacy [16,31-34] could reflect the increasing awareness of the impact of AI on this profession, as captured in this study. Chalasani et al [34] recently identified numerous applications for AI in pharmacy including adverse drug reaction detection, drug interaction identification, and dose recommendations. Varied perceptions may also be explained by the nature of the work conducted by each profession and the degree of direct and indirect patient care each profession provides. AHP skills are diverse, and the required knowledge, scope of practice, and competency standards are unique to each profession [69]. As a result, AI implementation strategies should be informed by profession-specific research to develop tailored approaches for each AHP profession rather than a one-size-fits-all approach.

Most of the participants in this study were not using AI in their current roles. The finding that AHPs who had used AI were more likely to feel prepared for AI when compared with those who had not used AI is consistent with other studies [24,34]. Chen et al [25] found that those who had used AI in the clinical setting had a better understanding of AI and were more positive about its potential application in health care. Even so, only 10% to 30% of health professionals worldwide have used AI in clinical practice [25]. Digital competence has been closely linked with professional confidence in previous studies exploring the introduction of new technology [70]. Professionals and managers recognize that technology such as electronic health records or telemedicine demands increased learning and new skills, which require time and exposure [71]. A lack of first-hand experience with AI can prevent AHPs from adapting and embracing AI in health provision. As the second largest workforce in health, AHPs have the potential to pioneer organizational change with AI implementation. Organizational efforts in digital transformation may be negatively affected if AHPs are not prepared adequately for AI adoption [16,25,42].

A lack of knowledge and first-hand experience with AI is emerging as a challenge for health professionals worldwide [23-25,66]. This study found that AHPs with lower AI knowledge (no knowledge to intermediate level knowledge) were less likely to think there will be a professional impact of AI when compared to AHPs with advanced level knowledge. Literature shows that most health professionals have a lack of basic AI knowledge [23,25] and most health professionals reported a lack of direct, hands-on experience with AI [24,25,66]. Education of AHPs about AI is urgently needed as a core implementation strategy for organizational adoption and preparedness [72].

### Perceptions on AI’s Purpose, Education, and Understanding of AI

In this study, AHPs expressed some skepticism about the purpose of introducing AI in health care and concern that it will reduce employment or remove valued clinical tasks. This is consistent with the reported concerns of medical officers in prior studies: AI replacing clinicians, taking over clinical tasks, or reducing the reliance on medical specialist experience [6,25,66,73]. Studies show that health care professionals are generally aware AI will have organizational and professional impacts that they are not yet prepared for which may threaten to undermine the benefits of AI before its implementation [24,26,74-77]. Despite this, AHPs in this study remain optimistic about the potential benefits of AI such as improving health care, clinical decision-making, and delivery of patient care, consistent with other studies [23,78,79]. These views are not dissimilar to those held in the previous digital health revolutions in which the rapid increase of the use of the internet and computers in health care delivery prompted an examination of the expectations, skills, and resources of users [80]. As the health service that has undergone considerable digital change in recent years, it is unsurprising that staff express trepidation based on their learned experience of digital health and apply these to AI. Consideration should be given to openly acknowledge, address, and monitor the impact of the unintended consequences of future AI implementation to help counter AHPs’ pessimism and inspire interest in this innovation [80].

When asked to describe their understanding of AI, AHP described AI as computers with intelligence, equipment with nonhuman qualities, human-like qualities, or as a robot, as found in prior literature [24]. With the absence of an agreed-upon definition of AI, it is not possible to assess how correct these descriptions are; however, they do demonstrate that AHPs are trying to understand AI. It is clear that the primary challenge facing AHPs implementing AI is a lack of knowledge and skills in AI, consistent with other findings [23,24]. This, combined with AHPs desire to learn shows this is the perfect time to address knowledge deficits. Targeted AI education and training would be highly beneficial considering most AHPs in this study had little to no knowledge about AI and reported little education, training, or experience with AI. Most AHPs desired AI training and indicated a preference for education about the application of AI in the health care setting, followed by ethical considerations of its use and general AI knowledge, consistent with prior findings [23,24]. Unsurprisingly, AHPs feel inadequately prepared or trained for AI with the current lack of education or any competency framework available for health professionals to develop skills in digital health [69,81]. Incorporation of AI education into professional, tertiary, and workplace training is crucial to overcome the barriers that may otherwise limit the adoption of AI in health care [23,25]. Strategies identified to help address critical knowledge and experience deficits of clinicians include professional organization position statements, professional accreditation, digital literacy embedded in both undergraduate and postgraduate tertiary education, multidisciplinary team learning, and specialist digital health career pathways [66,69,81].

### Opportunities for AI in AH

Our findings suggest that AHPs are well placed to contribute to the co-design of AI applications in clinical settings to increase the use of AI tools to improve patient and system outcomes. Indeed, approximately a third of respondents identified ideas for the application of AI in AH. Ideas are predominantly related to nonclinical, administrative tasks, and to support patient assessment to improve efficiencies and increase the clinical time for direct patient care. It is worth noting that ideas were generated by some clinicians with a lack of knowledge and experience with AI, but who were generally optimistic about the future impact of AI. This shows that industry and academic partnerships with clinicians should not be limited to engagement with those with high AI literacy as clinicians across all knowledge levels may still be able to identify relevant AI opportunities.

### Limitations and Future Directions

A key strength of this study was the relatively high response rate (22.1%) of the survey leading to an estimated margin of error of 0.045 and broad representation across varied AHP professions. However, we still cannot be certain that the sample is representative of the whole AHP population.

Key limitations of this study include, first, responders, compared to nonresponders, may have had a specific interest in AI, either negative or positive, therefore AHPs that are not interested or aware of AI could be underrepresented. Second, the survey collection tool named eleven GCHHS AHP departments, which may have led to the lack of participation from unnamed professions, for example, music therapists, and limits the applicability of the findings to the broader range of AH professions. Third, clinical measurements and medical imaging [16,66,79] clinicians were not well represented in the data despite efforts to engage and recruit; therefore, targeting these populations may be a consideration in future studies. Fourth, the conduct of the survey within a single hospital and health service study setting may limit the generalizability of the findings to AHPs in other settings. Fifth, the survey did not meet the ideal minimum Cronbach α for internal consistency for factor two: preparedness for AI, likely due to the small number of items contributing to the factor. Further research should explore the key barriers and enablers of implementing AI in health care from the AHP perspective, to inform AI implementation strategies and facilitate the adoption of AI.

### Clinical Implications

This study highlights that AHPs perceive they are unprepared for AI implementation within their health care setting. As the second largest workforce in health [42], the preparation of AHPs should be a priority given the rate at which AI is developing in the health care sector. This should include targeted education and training, along with first-hand experience with AI to maximize readiness for the coming widespread adoption and implementation of AI across health care.

With a lack of external training providers and limited clinician time available outside official duty hours, health care organizations should consider how to mobilize the workforce to learn and use new AI technologies tailored to the different needs of the professional groups [16]. Organizations should consider collaborating with AHP and digital industry experts on identifying and exploring opportunities for AI in health care, regardless of digital knowledge and readiness.

### Conclusion

AH, the second largest workforce in health, has untapped potential to help pioneer AI implementation in health care. A lack of workforce AI knowledge or skills was identified as a potential key barrier to implementation. Targeted education, training, and hands-on experience with AI should be prioritized for AHP to support the implementation of the rapidly emerging digital revolution. Further research is required to more deeply understand the barriers and enablers of AI implementation from the perspective of the AHP to tailor education and inform workforce readiness strategies to drive change and lead innovation. Industry and academic partnerships with clinicians should not be limited to AHPs with high AI literacy as clinicians across knowledge levels can identify many opportunities for AI in health care.

## Appendix

Multimedia Appendix 1 Univariable linear regressions: allied health professional perceptions of professional impact of AI and preparedness for AI (N=231) table. AI: artificial intelligence.

## Abbreviations

AAMLS: approximate adjusted mean Likert Scale
AH: allied health
AHP: allied health professional
AI: artificial intelligence
AMFS: adjusted mean factor score
CFA: confirmatory factor analysis
CFI: comparative fit index
CVSR: critical value of studentized range
GCHHS: Gold Coast Hospital and Health Service
RMSEA: root-mean-square error of approximation
SHAIP: Shinners Artificial Intelligence Perception
SRMR: standardized root-mean-squared residual
STROBE: Strengthening the Reporting of Observational Studies in Epidemiology
TLI: Tucker-Lewis index

## Data Availability

The datasets generated or analyzed during this study are available from the corresponding author upon reasonable request.
