# Supplementary material for: Allied Health Professionals’ Perceptions of Artificial Intelligence in the Clinical Setting: Cross-Sectional Survey
Source: JMIR Form Res. 2024 Dec 30;8:e57204. doi: 10.2196/57204 (PMC11730220; doi:10.2196/57204)
Supplement: Multimedia Appendix 1 [file formative_v8i1e57204_app1.docx]

**Table S1.** Univariable linear regressions: allied health professional perceptions of *professional impact of AI* ^a^ and *preparedness for AI* ^a^ (n=231).

| Variable | Category | Factor one: *Professional impact of AI* ^a^ | | | | Factor two: *Preparedness for AI* ^a^ | | | |
| --- | --- | --- | --- | --- | --- | --- | --- | --- | --- |
|  |  | Mean Likert Score | Mean factor Score | *P* value | Overall  *P* value | Mean Likert Score | Mean factor Score | *P* value | Overall *P* value |
|  |  |  |  |  |  |  |  |  |  |
| **Age** | 18 to 30 | *2.55* | -.038 | * | *.114* | *3.99* | -.018 | * | .862 |
|  | 31 to 40 | *2.59* | -.001 | .684 |  | *3.93* | -.015 | .978 |  |
|  | 41 to 50 | *2.53* | -.085 | .659 |  | *3.91* | -.012 | .967 |  |
|  | 51 to 60 | *2.94* | .247 | .023 |  | *4.00* | .061 | .616 |  |
|  | 61 to 70 | *2.38* | -.172 | .629 |  | *4.42* | .334 | .316 |  |
| **Gender** | Women | *2.65* | .037 | .030 | *.089* | *3.92* | -.325 | .169 | .225 |
|  | Men | *2.42* | -.168 | * |  | *4.11* | .129 | * |  |
|  | Prefer not to say | *2.42* | -.124 | .910 |  | *4.5* | .495 | .453 |  |
| **Profession** | Audiology | *2.5* | -.011 | .146 | *<.001* | *4.06* | .099 | .820 | .748 |
|  | Dietetics | *2.60* | .057 | .001 |  | *3.89* | .050 | .886 |  |
|  | Medical imaging | *2.39* | -.194 | .334 |  | *3.79* | -.154 | .429 |  |
|  | Occupational Therapy | *2.70* | .108 | <.001 |  | *4.10* | .124 | .511 |  |
|  | Other | *2.83* | .319 | .065 |  | *4.5* | .503 | .332 |  |
|  | Pharmacy | *2.17* | -.358 | * |  | *3.93* | .027 | * |  |
|  | Physiotherapy | *2.92* | .235 | <.001 |  | *3.89* | -.114 | .342 |  |
|  | Podiatry | *2.61* | -.059 | .323 |  | *4.00* | .286 | .521 |  |
|  | Psychology | *2.59* | -.044 | .066 |  | *4.15* | .120 | .683 |  |
|  | Social work | *2.94* | .215 | <.001 |  | *3.79* | -.192 | .186 |  |
|  | Speech pathology | *2.51* | -.066 | .043 |  | *4.14* | .038 | .952 |  |
|  | Unknown | *2.28* | -.303 | .855 |  | *4.00* | -.134 | .692 |  |
| **Role** | Clinical Informatics/technology | *2.13* | -.531 | * | *.059* | *4.00* | .066 | * | .893 |
|  | Clinician | *2.65* | .046 | .018 |  | *3.94* | -.021 | .776 |  |
|  | Educator/clinical facilitator | *2.53* | -.103 | .205 |  | *3.87* | .123 | .894 |  |
|  | Governance | *2.33* | -.278 | .665 |  | *3.67* | -.063 | .862 |  |
|  | Manager | *2.38* | -.181 | .195 |  | *4.06* | .114 | .889 |  |
|  | Researcher/academic | *2.17* | -.476 | .877 |  | *3.92* | .042 | .957 |  |
|  | Unknown | *2.5* | -.177 | .427 |  | *4.67* | .552 | .393 |  |
| **Years of experience with ieMR** ^b^? | 0 | *2.22* | -.232 | * | *.006* | *3.77* | -.197 | * | .106 |
|  | 0-1 | *2.35* | -.191 | .906 |  | *3.73* | -.544 | .425 |  |
|  | 1-2 | *2.31* | -.245 | .967 |  | *3.90* | -.019 | .662 |  |
|  | 2-3 | *2.67* | .103 | .304 |  | *3.89* | -.165 | .937 |  |
|  | 3-4 | *2.54* | -.019 | .499 |  | *4.07* | .105 | .450 |  |
|  | 4+ | *2.68* | .044 | .370 |  | *3.96* | .047 | .532 |  |
|  | Unknown | *4.00* | 1.126 | .005 |  | *4.17* | .139 | .581 |  |
| **AI** ^a^ **knowledge** | No knowledge | *2.84* | .235 | <.001 | *<.001* | *3.91* | -.057 | .187 | .367 |
|  | Beginner understanding | *2.59* | -.013 | .003 |  | *3.98* | .026 | .269 |  |
|  | Intermediate understanding | *2.49* | -.158 | .025 |  | *3.81* | -.146 | .123 |  |
|  | Advanced knowledge | *1.63* | -.733 | * |  | *4.27* | .365 | * |  |
| **Use of AI** ^a^ | Yes | *2.19* | -.409 | .002 | *.004* | *3.67* | -.163 | .243 | .050 |
|  | Unsure | *2.45* | -.116 | .100 |  | *3.70* | -.237 | .024 |  |
|  | No | *2.66* | .051 | * |  | *4.02* | .053 | * |  |
| **Want education on AI** ^a^**?** | Yes | *2.54* | -.070 | .043 | *<.001* | *3.97* | -.001 | .456 | .420 |
|  | Unsure | *2.74* | .157 | .845 |  | *3.96* | .038 | .392 |  |
|  | No | *2.74* | .185 | * |  | *3.75* | -.123 | * |  |
|  | Unknown | *5.00* | 1.913 | .001 |  | *5.00* | .970 | .115 |  |

*Used as comparator group, ^a^ artificial Intelligence, ^b^ integrated electronic medical records
